# The clinical phenotype with gastrostomy and abdominal wall infection in a pediatric patient with Takenouchi-Kosaki syndrome due to a heterozygous c.191A > G (p.Tyr64Cys) variant in *CDC42*: a case report

**DOI:** 10.3389/fgene.2023.1108852

**Published:** 2023-06-06

**Authors:** Aleksandra Szczawińska-Popłonyk, Natalia Popłonyk, Magdalena Badura-Stronka, Jerome Juengling, Kerstin Huhn, Saskia Biskup, Bartłomiej Bancerz, Jarosław Walkowiak

**Affiliations:** ^1^ Department of Pediatric Pneumonology, Allergy and Clinical Immunology, Institute of Pediatrics, Karol Jonscher University Hospital, Poznań University of Medical Sciences, Poznań, Poland; ^2^ Student Scientific Society, Poznań University of Medical Sciences, Poznań, Poland; ^3^ Centers for Medical Genetics Genesis, Poznań, Poland; ^4^Chair and Department of Medical Genetics, Poznań University of Medical Sciences, Poznań, Poland; ^5^ Zentrum Fur Humangenetik Tübingen, Tübingen, Germany; ^6^ CeGaT GmbH, Tübingen, Germany; ^7^Department of Pediatric Gastroenterology and Metabolic Diseases, Institute of Pediatrics, Karol Jonscher University Hospital, Poznań University of Medical Sciences, Poznań, Poland

**Keywords:** Takenouchi-Kosaki syndrome, Cdc42, c.191A>G variant, antibody deficiency, macrothrombocytopenia, neurodevelopmental delay

## Abstract

The *CDC42* (cell division cycle homolog 42) gene product, Cdc42 belongs to the Rho GTPase family which plays a pivotal role in the regulation of multiple cellular functions, including cell cycle progression, motility, migration, proliferation, transcription activation, and reactive oxygen species production. The Cdc42 molecule controls various tissue-specific functional pathways underpinning organogenesis as well as developmental integration of the hematopoietic and immune systems. Heterozygous c.191A>G (p.Tyr64Cys) pathogenic variants in *CDC42* cause Takenouchi-Kosaki syndrome characterized by a spectrum of phenotypic features comprising psychomotor developmental delay, sensorineural hearing loss, growth retardation, facial dysmorphism, cardiovascular and urinary tract malformations, camptodactyly, accompanied by thrombocytopenia and immunodeficiency of variable degree. Herein, we report a pediatric patient with the Takenouchi-Kosaki syndrome due to a heterozygous p.Tyr64Cys variant in *CDC42* manifesting as a congenital malformation complex accompanied by macrothrombocytopenia, poor specific antibody response, B and T cell immunodeficiency, and low serum immunoglobulin A level. We also suggst that feeding disorders, malnutrition, and a gastrointestinal infection could be a part of the phenotypic characteristics of Takenouchi-Kosaki syndrome supporting the hypothesis of immune dysregulation and systemic inflammation occurring in the p.Tyr64Cys variant in *CDC42.*

## Introduction

The *Cell Division Cycle 42* (*CDC42*) gene encodes a Cdc42 molecule which is a member of the family of Rho GTPases belonging to the Ras superfamily of small GTPases. The Rho-family GTPases are characterized by a multiplicity of physiological functions influencing crucial developmental signals in cell cycle regulation. These biological processes include the establishing and controlling of the cell actin cytoskeleton, vesicle trafficking, cell polarity, proliferation, motility and migration, transcription activation, reactive oxygen species production, and malignant transformation (Ueyama, 2019; Lauri et al., 2021). The Cdc42 protein plays a fundamental role in cell biology and is implicated in a wide array of physiologically pivotal, tissue-specific activities. Cdc42-dependent cytoskeletal reorganization and cell polarity are essential for cardiac organogenesis, tubulogenesis of the pancreas, kidney, lung, and salivary and mammary glands, and differentiation of keratinocytes in the skin and hair follicles (Melendez et al., 2011; Liu et al., 2013). In the central nervous system (CNS), Cdc42 is a positive regulator of its development through neurite initiation, axon growth, branching, myelination and specification, neuronal migration as well as neuronal polarity (Melendez et al., 2011; Liu et al., 2013). Cdc42 coordinates actin turnover and maintenance of actin structures stability in inner ear hair cells and the polarization of the sensory organ of the cochlea which determines the hearing function. The processes of cell polarization and actin microtubule dynamics also play a role in eye morphogenesis and photoreceptor differentiation (Ueyama et al., 2014).

Cdc42 participates in a wide spectrum of immune functions related to hematopoiesis and immune homeostasis as well as effector mechanisms of the innate and adaptive immune responses. In the bone marrow, Cdc42 regulates the multilineage development of blood progenitors and their egress from the bone marrow to the periphery. While Cdc42 is involved in the transcriptional program, it plays an important role in the differentiation of stem and progenitor cells, thus influencing the tight balance between myelopoiesis and erythropoiesis (Mulloy et al., 2010; Nayak et al., 2013), and thereby contributing to the immune system regulation and activation. In neutrophil granulocytes, the Cdc42 molecule, through the cytoskeleton rearrangement, controls migration, activation, degranulation, and reactive oxygen species formation thus influencing pathogen killing efficiency (Tackenberg et al., 2020). The migratory function of dendritic cells upon antigen stimulation and homing to the draining lymph nodes is also Cdc42 activity-depending, reflecting the Cdc42 substantial contribution to the initiation of the adaptive response of T CD4^+^ helper and CD8^+^ cytotoxic/suppressor lymphocytes (Luckashenak et al., 2013). The Cdc42 GTPase is an essential coordinator of B lymph cell development, adhesion, motility, antigen and mitogen-driven B cell receptor activation, cognate interaction with T lymph cells, and differentiation into antibody-producing plasma cells (Song et al., 2014; Burbage et al., 2015; Gerasimcik et al., 2015). It has also been shown that Cdc42 plays an important role in T cell thymopoiesis, proliferation, and survival (Smits et al., 2010).

Referring to the multiplicity of key regulatory functions of the Cdc42 GTPase in human biology, governing morphogenesis and functional homeostasis, the growing spectrum of clinical syndromes and pathogenic variants in *CDC42* underpinning the diverse phenotypes have been reported. The patients with a germlin heterozygous missense c.191A>G (p.Tyr64Cys) variant demonstrated a range of congenital phenotypic features termed as Takenouchi-Kosaki syndrome (TKS). The syndrome is characterized by facial dysmorphism, developmental delay, sensorineural hearing loss accompanied by macrothrombocytopenia which compose a universal clinical phenotype observable in all eight hitherto described TKS patients (Takenouchi et al., 2015; Takenouchi et al., 2016; Martinelli et al., 2018; Motokawa et al., 2018; Uehara et al., 2019; Bucciol et al., 2020; Ishikawa et al., 2021; Santoro et al., 2021). Short stature (Takenouchi et al., 2015; Takenouchi et al., 2016; Martinelli et al., 2018; Bucciol et al., 2020; Ishikawa et al., 2021; Santoro et al., 2021), failure to thrive (Takenouchi et al., 2015; Takenouchi et al., 2015; Motokawa et al., 2018; Bucciol et al., 2020; Ishikawa et al., 2021; Santoro et al., 2021) with camptodactyly (Takenouchi et al., 2015; Takenouchi et al., 2016; Martinelli et al., 2018; Motokawa et al., 2018; Bucciol et al., 2020; Ishikawa et al., 2021; Santoro et al., 2021), structural abnormalities of the brain (Takenouchi et al., 2015; Takenouchi et al., 2016; Martinelli et al., 2018; Motokawa et al., 2018; Uehara et al., 2019; Bucciol et al., 2020; Santoro et al., 2021), and antibody deficiency (Martinelli et al., 2018; Motokawa et al., 2018; Bucciol et al., 2020; Ishikawa et al., 2021) represent not constant but frequent findings belonging to the symptomatology in TKS. Herein, we report an another patient presenting with distinctive symptoms of TKS (OMIM #616737) due to the heterozygousp.Tyr64Cys variant in *CDC42* and the clinical phenotype with congenital malformations, immunodeficiency, and gastrointestinal disorders.

## Case report

### The patient

The male patient was born to a non-consanguineous couple from the first pregnancy complicated by premature uterine contracions and spontaneous vaginal delivery at the 38th week of gestational age (WGA), with an Apgar score of 8 points. His anthropometric parameters at birth were the following: weight 3.52 kg [+0.60 standard deviation score (SDS)], length 54 cm (+2.00 SDS), and head circumference 37 cm (+1.25 SDS). The perinatal period was complicated by respiratory distress syndrome due to congenital pneumonia resulting from ascending intrauterine infection and he required non-invasive ventilation support (nCPAP) and antibiotic therapy with ampicillin and gentamycin for 10 days; subsequently, intermittent low-flow supplemental oxygen therapy was needed by the 33rd day of life. He presented features suggesting an underlying genetic syndrome consisting of facial dysmorphism, low-set ears, axial hypotonia with peripheral spasticity, camptodactyly, and cutaneous syndactyly of toes. The abdominal ultrasound examination demonstrated a duplex pelvicalyceal system of the right kidney and in echocardiography, an atrial septal defect (ASD) was found. In the cerebral magnetic resonance imaging (MRI), widening of the supratentorial ventricular system, in particular in the region of occipital and temporal horns of lateral ventricles caused by narrowing of the periventricular white matter as well as hypoplastic cerebellar vermis were found. Due to the poor sucking reflex in a newborn, making the initiation of breastfeeding and bottle-feeding unsuccessful despite the oral motor stimulation and frenotomy, feeding by a nasogastric tube to secure the nutrition was recommended. However, feeding intolerance and inappropriate weight gain velocity were observable. The infant’s diet was modified and he received an amino acid-based formula with nutritional, caloric, and thickening supplementation yet failure to thrive was disturbing the patient’s growth and weight gain, and achieving developmental milestones. At the age of 3 months, the patient’s weight was 5.15 kg (−0.80 SDS), length 60 cm (+2.30 SDS), and head circumference 37.5 cm (−1.50 SDS). At that time, the patient was admitted to the department of pediatric gastroenterology and metabolic diseases of our tertiary care university hospital to provide enteral feeding, a balloon-assisted endoscopic percutaneous gastrostomy (PEG) feeding tube was inserted using a push method. The procedure was complicated by an infection of the skin, abdominal wall, and intraperitoneal connective tissue of the anterior gastric wall, from which *Staphylococcus aureus* and *Enterococcus faecalis* were cultured, and hence, intensive intravenous antibiotic therapy with meropenem and replacement of PEG were necessary.

At the age of 9 months, the patient was admitted to our university pediatric immunology unit for immunodiagnostics. He presented with proportionate growth retardation, his body mass stood at 6.9 kg (−1.25 SDS), length 70 cm (−1.50 SDS), head circumference 42,5 cm (SDS −1.62), and the weight-for-length ratio below the third percentile (−2.25 SDS), bilateral profound sensorineural hypoacusis, axial hypotonia with peripheral spasticity, sloping forehead and low hairline, facial dysmorphism with an oblique setting of palpebral fissures, nasal tip pointed upwards, downturned corners of the lips, arched palate, low-set ears, camptodactyly and cutaneous syndactyly of toes, micropenis, and cryptorchidism. Developmental psychomotor delay was observable as the boy showed poor facial expression, did not make any sounds, get to a sitting position by himself nor sit without support. The clinical evaluation, biochemical laboratory tests, and ultrasound imaging assessment did not show features of lymphoproliferation, hemophagocytic lymphohistiocytosis (HLH), or bleeding disorders. The immunodiagnostic workup revealed macrothrombocytopenia, lymphopenia, low serum immunoglobulin A level, poor specific antibody response, and impairment of humoral and cellular immunity. An in-depth flow cytometric immunophenotyping showed abnormalities within the B and T lymph cell compartment primarily disrupted T cell development with low T CD4^+^ helper cell, recent thymic emigrant numbers, and naïve T CD45RA + cell to memory T CD45RO + cell ratio. These were accompanied by low numbers of B cells and a decline in B cell memory development. The immunological diagnostics performed on the patient studied is displayed in Table 1 (Part A). Due to the patient’s history pointing to recurrent infections, microbiology studies were performed. The infectious workup targeted at airway and blood-borne viral and bacterial pathogens, proved negative as shown in Table 1 (part B).

**TABLE 1:** The immunological workup with antibody-mediated response and peripheral blood flow cytometric immunophenotyping (Part A) and the infectious workup (Part B) in the patient with p.Tyr64Cys variant in *CDC42*.

A. Immunological workup		
Antibody response	Results	Reference values
Immunoglobulins		
IgG	474 mg/dL	350–1180 mg/dL
IgA	13 mg/dL	36–165 mg/dL
IgM	30 mg/dL	30–104 mg/dL
IgE	<2 kU/L	>2 kU/L
IgG subclasses		
IgG1	296 mg/dL	194–842 mg/dL
IgG2	34 mg/dL	22–300 mg/dL
IgG3	92 mg/dL	19–85 mg/dL
IgG4	18 mg/dL	5-78 mg/dL
Antigen-specific antibodies		
Anti-Diphtheria toxoid IgG	0.11 IU/mL	>1.0 IU/mL
Anti-Tetanus toxoid IgG	0.66 IU/mL	>1.0 IU/mL
PB lymphocyte immunophenotyping		
Full PB count		
WBC	4,160cc	4,000–20,000cc
Lymphocytes CD45+/SSC low	58.0%, 2,400cc	57.0-83.6%, 4000–8,600cc
B cell compartment		
B CD19+	16.0%, 400cc	15.7–34.1%, 700–2,800cc
Transitional B CD19+CD38+IgM++	47.2%, 189cc	7.2–19.7%, 100–300cc
Mature naїve B CD19+CD27-IgD+	89,3%, 357cc	85.5–93.4%, 600–2,590cc
Non-switched memory B (MZL) CD19+CD27+IgD+	1.9%, 8cc	2.8–7.4%, 30–120cc
Switched memory B CD19+CD27+IgD-	2.3%, 9cc	1.6–7.0%, 20–140cc
Immature B CD19+CD21lo	34.2%, 137cc	6.2–20.3%, 70–290cc
Immature activated B CD19+CD38loCD21lo	3.4%, 14cc	0.4–3.3%, 0–50cc
Plasmablasts CD19+CD38++IgM-	0.8%, 3cc	0.2–4.0%, 0–60cc
T cell compartment		
T CD3+	51.0%, 1254cc	49.0–95.0%, 1,400–11,500cc
T helper CD3+CD4+	24.0%, 593cc	27.0–81.0%, 1,000–7,200cc
T suppressor/cytotoxic CD3+CD8+	23.0%, 561cc	10.0–35.0%, 200–5,400cc
CD4+/CD8+	1.1	1.5–2.5
CD45RA+/CD45RO+	1.0	>1.0
Recent thymic emigrants CD3+CD4+CD45RA+CD31+	48.1%, 285cc	65.0–90.0%, 800–6,200cc
Naïve T helper CD3+CD4+CD45RA+CD27+	48.1%, 285cc	77.0–97.0%, 800–7,600cc
Central memory T helper CD3+CD4+CD45RA-CD27+	46.1%, 274cc	2.0–59.0%, 100–1,300cc
Effector memory T helper CD3+CD4+CD45RA-CD27-	4.8%, 28cc	0.1–1.0%, 1–72cc
Terminally differentiated memory T helper CD3+CD4+CD45RA+CD27-	1.0%, 6cc	0.0–7.3%, 0–400cc
Follicular CXCR5+ T helper CD3+CD4+CD45RO+CD185+	19.7%, 56cc	9.0–47.0%, 15–110cc
Regulatory T helper CD3+CD4+CD25++CD127-	2.6%, 15cc	4.0–18.0%, 60–740cc
Naïve T suppressor/cytotoxic CD3+CD8+CD27+CD197+	26.0%, 146cc	31.0–100%, 150–3200cc
Central memory T suppressor/cytotoxic CD3+CD8+CD45RA-CD27+CD197+	5.4%, 30cc	0.1–13.0%, 2–150cc
Effector memory T suppressor/cytotoxic CD3+CD8+CD45RA-CD27-CD197-	33.8%, 180cc	1.0–100%, 8–1,400cc
Terminally differentiated T suppressor/cytotoxic CD3+CD8+CD45RA+CD27-CD197-	11.8%, 66cc	5.0–78.0%, 17–2,800cc
NK cells		
NK CD3-CD45+CD16+CD56+	25.0%, 628cc	2.0–36.0%, 61–510cc
Lymphocyte stimulation tests		
PHA	3,7261 cpm	>160,000 cpm
PHA stimulation index	142.2	>65.0
Anti-CD3	6,6818 cpm	>15,000 cpm
Anti-CD3 stimulation index	255.0	>60.0
Pansorbin cells	4,399 cpm	>2,000 cpm
Pansorbin cells stimulation index	16.8	>5.0
Complement		
C3	148 mg/dL	90–180 mg/dL
C4	34 mg/dL	10–40 mg/dL
CH50	156 EqU/mL	70–187 EqU/mL
B. Infectious workup		
Blood		Results
Viral panel Cytomegalovirus-DNA, Epstein-Barr virus-DNA, Adenovirus-DNA, Herpes simplex virus 1 and 2-DNA, Human Herpes virus 6 and 7-DNA, Enterovirus-RNA, Human Parechovirus-RNA, Varicella zoster virus-DNA, Human Parvovirus B19-DNA, Hepatitis B-DNA, Hepatitis C-DNA		All (-)
Bacterial panel Streptococcus pneumoniae-DNA, Neisseria meningitidis-DNA, Haemophilus influenzae-DNA		All (-)
Airways		
Airway aspirate Influenza virus A, A H1N1, B-RNA, Rhinovirus-RNA, Coronavirus NL63, 229E, OC43, HKU1-RNA, panel Parainfluenza virus 1,2,3,4-RNA, Human metapneumovirus A, B-RNA, Respiratory syncytial virus A, B-RNA, Enterovirus-RNA, Parechovirus-RNA, Bocavirus-DNA, Mycoplasma pneumoniae-DNA, Chlamydophila pneumoniae-DNA, Staphylococcus aureus-DNA, Streptococcus pneumoniae-DNA, Haemophilus influenzae-DNA		All (-)

**FIGURE 1:**
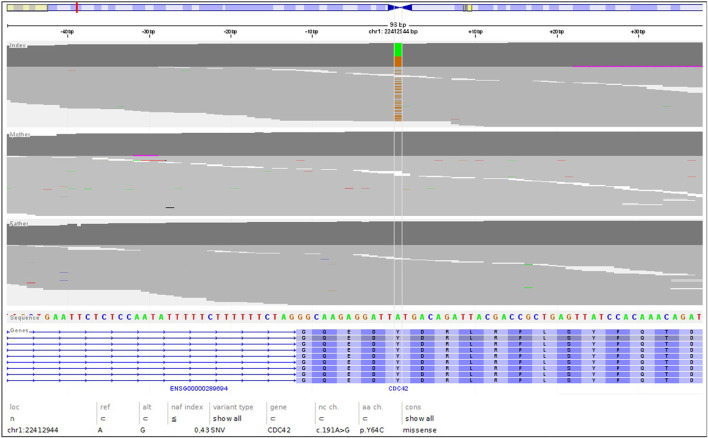
The NGS reads of the trio, highlighting the *de novo* nature of the c.191A>G; pTyr64Cys variant.

**FIGURE 2:**
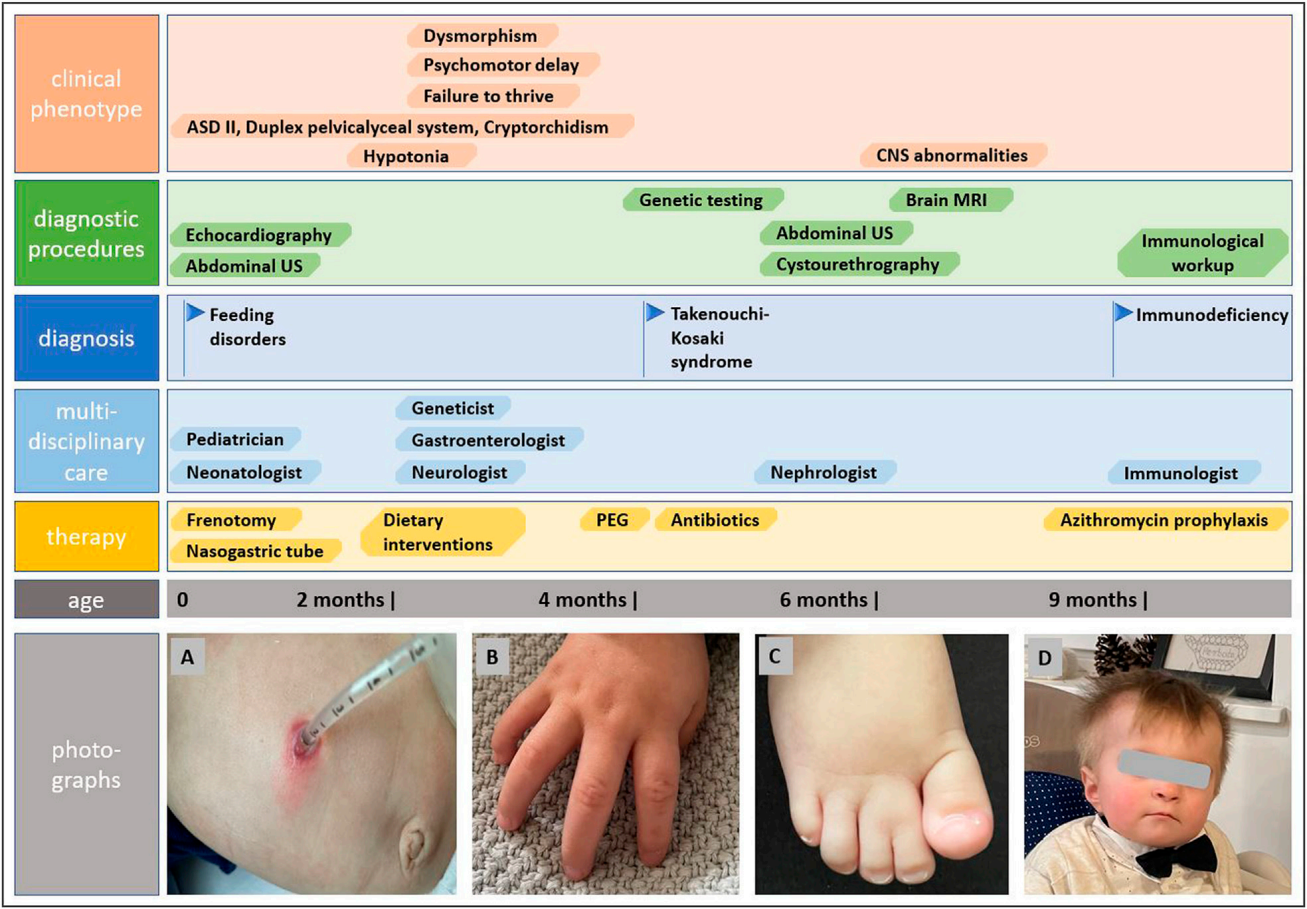
Timeline showcasing the course of the disease, establishing the diagnosis of Takenouchi-Kosaki syndrome and the multidisciplinary approach. Photographs show gastrostomy and abdominal wall infection **(A)**, camptodactyly **(B)**, syndactyly of toes **(C)**, and distictive facial gestalt **(D)**.

The genetic analysis using exome technique on trio was performed. In the patient, it revealed the c.191A>G (p.Tyr64Cys) (NM_001791.4) variant in the *CDC42* gene. The presence of the variant was confirmed in the proband by Sanger sequencing and excluded in both parents, indicating that the variant occurred *de novo* in a heterozygous state (Figure 1). The p.Tyr64Cys variant in conjunction with the patient’s clinical phenotype is causative for TKS (Takenouchi et al., 2015; Takenouchi et al., 2016; Martinelli et al., 2018; Motokawa et al., 2018; Uehara et al., 2019; Bucciol et al., 2020; Ishikawa et al., 2021; Santoro et al., 2021). The summary of the clinical phenotypic features, course of the disease including gastroenterological problems, diagnostic procedures uncovering congenital anomalies and immunodeficiency, as well as implemented multidisciplinary care are displayed in Timeline (Figure 2).

## Methods

### The molecular genetic analysis

Trio whole exome sequencing (WES) analysis was performed on the proband and his parents.

Starting from EDTA blood, genomic DNA was isolated according to the manufacturers’ instructions using QIAamp DNA Blood Maxi Kit on a QiaSymphony instrument (Qiagen, Hilden, Germany). DNA quantity and quality are determined using Qubit®uFluorometer and NanoDrop ND-8000 (Thermo Fisher Scientific, Dreieich, Germany). Sequencing libraries were prepared for each sample from 50 ng DNA using the Twist enrichment workflow (Twist Bioscience, San Francisco, CA, United States) and a custom-design enrichment probe set (CeGaT ExomeXtra V2). Library preparation and capture was performed according to the manufacturer’s instructions and paired-end sequencing was performed on a NovaSeq6000 instrument (Illumina, San Diego, CA, United States) with 2 × 100 base pairs (bp) read length. Sequence data were processed with Illumina bcl2fastq2. Adapter sequences were removed with Skewer and the sequences obtained, were aligned to the human reference genome (hg19) with the Burrows Wheeler aligner (BWA mem). Sequences that could not be clearly assigned to a genomic position were removed, as were sequence duplicates that were probably due to amplification (internal software). Copy number variations (CNV) were computed on uniquely mapping, non-duplicate, high quality reads using an internally developed method based on sequencing coverage depth. Briefly, we used reference samples to create a model of the expected coverage that represents wet-lab biases as well as intersample variation. CNV calling was performed by computing the sample’s normalized coverage profile and its deviation from the expected coverage. Genomic regions were called as variant if they deviate significantly from the expected coverage. Sequence variants (single nucleotide exchanges and short insertions/deletions) were determined from the remaining high-quality sequences (CeGaT StrataCall). Resulting variants were annotated with population frequencies from gnomAD (2.1/3.1) and an internal database (CeGaT), factoring in external databases (e.g., HGMD, ClinVar), and with transcript information from Ensembl, RefSeq, Gencode, and CCDS. All variants were manually assessed before inclusion in the final report, and classified and reported based on ACMG/ACGS-2020v4.01 guidelines (Richards et al., 2015).

### Flow cytometric peripheral blood (PB) lymph cell immunophenotyping

Cells were labeled with the following murine fluorochrome-stained monoclonal antibodies: anti-CD45 FITC (fluorescein isothiocyanate), anti-CD14 PE (phycoerythrin), anti-CD19 PE, anti-CD19 PerCP (peridinin chlorophyll protein), anti-IgM FITC, anti-IgD FITC, anti-CD38 APC (allophycocyanin), anti-CD27 PE, anti-CD21 FITC, as well as anti-CD3 FITC, anti-CD4 FITC, CD45RA FITC, CD127 FITC, CD185 FITC, anti-CD8 PE, anti-CD16^+^CD56 PE, CD25 PE, CD31 PE, CD45RO PE, anti-CD3 PerCP, CD197 PerCP, anti-CD4 APC and anti-CD8 APC (all Beckton-Dickinson Biosciences, United States). The acquisition of cells and analysis was carried out with the use of the flow cytometer FACSCanto and FACSDiva software (Beckton-Dickinson, United States). With sequential gating on biparametric scattering CD45^+^CD14-lymphocytes, the following lymphocyte subpopulations were identified:

- CD19^+^ B cells, immature CD19^+^CD21lo, immature activated CD19^+^CD38loCD21lo, transitional CD19^+^CD38hisIgMhi, non-switched memory CD19^+^CD27+sIgD+, switched memory CD19^+^CD27+IgD- B cells, and CD19^+^CD38hisIgM-plasmablasts
- CD3^+^ T cells, CD3^+^CD4^+^ T helper cells, CD3^+^CD4^+^CD31^+^CD45RA + recent thymic emigrants, naïve CD3^+^CD4^+^CD27^+^CD45RA+, regulatory CD3^+^CD4^+^CD25++CD27^−^, central memory CD3^+^CD4^+^CD27^+^CD45RO+, effector memory CD3^+^CD4^+^CD27^−^CD45RO+, terminally differentiated CD3^+^CD4^+^CD27^−^CD45RA+, follicular CD3^+^CD4^+^CD185+CD45RO+, and regulatory CD3^+^CD4^+^CD45RO+CD127-CD25++ T helper cells. Among CD3^+^CD8^+^ cytotoxic T cells, the following subsets were distinguished: naïve CD3^+^CD8^+^CD197+CD27^+^CD45RA+, central memory CD3^+^CD8^+^CD197+CD27^+^CD45RO+, effector memory CD3^+^CD8^+^CD197-CD27^−^CD45RO+, and terminally differentiated CD3^+^CD8^+^CD197-CD27^−^CD45RA + cells.
- CD3^−^CD16^+^CD56^+^ NK cells.

The relative values of PB lymphocytes, the B, T, and NK cells of the total lymphocyte population as well as B and T cell subsets were calculated. The absolute counts of all cell subsets were calculated from the PB leukocyte counts. A comparative analysis was done with the reference cut-off values of B and T cell subsets for pediatric populations of different age groups (Piątosa et al., 2010; Schatorje et al., 2012).

## Discussion

Referring to the heterogeneity of clinical manifestations and the complexity of mutual phenotype-genotype interrelations in hitherto reported alterations in *CDC42*, it must be highlighted that establishing the definitive diagnosis of a *CDC42*- related syndrome, including TKS, is challenging for clinicians. The phenotypic features of TKS related to the p.Tyr64Cys variant in *CDC42* comprise psychomotor developmental delay, intellectual disability, dysmorphism, sensorineural hearing loss, cardiac and genitourinary malformations, and macrothrombocytopenia (Takenouchi et al., 2015; Takenouchi et al., 2016; Martinelli et al., 2018; Motokawa et al., 2018; Uehara et al., 2019; Bucciol et al., 2020; Ishikawa et al., 2021; Santoro et al., 2021), consistent also with the herein reported patient. The summary of phenotypic features of patients with TKS due to the c.191A>G; p.Tyr64Cys variant in *CDC42* are summarized in Table 2. Noteworthy, in a single patient with the same p.Tyr64Cys variant, localized in the switch II domain of the *CDC42* gene, affecting Cdc42 binding to multiple effectors and regulators, and causing TKS (Martinelli et al., 2018), immune dysregulation with autoinflammation was observable. It was characterized by interleukin (IL)-6, IL-18, IL-18 binding protein (BPa), and CXCL9 chemokine hypercytokinemia accompanied by myelofibrosis (Bucciol et al., 2020). This phenomenon reflects the clinical intersubject variability within the p.Tyr64Cys variant group and possible overlap with C-terminal variants in the *CDC42* gene and highlights that the p.Tyr64Cys variant does support the hypothesis that the gene is associated with distinct diseases. The clinical phenotypes related to C-terminal variants are characterized by severe neonatal-onset dyshematopoiesis and immune dysregulation including autoinflammation, rash, and HLH (NOCARH syndrome) (Gernez et al., 2019; Lam et al., 2019; He et al., 2020; Coppola et al., 2022) with a chronic excess of the inflammatory cytokine, IL-18 as a hallmark of these disorders (Miyazawa and Wada, 2022; Shimizu et al., 2022). A similar phenotype distinguished by the hyperinflammatory state, full-blown HLH, and hitherto not reported, development of non-Hodgkin lymphoma has also been described by our group in the patient with a novel p.Cys81Tyr variant (Szczawińska-Popłonyk et al., 2020).

**TABLE 2:** The summary of clinical symptomatology presented by patients with the same p.Tyr64Cys variant in *CDC42*.

Clinical workup of takenouchi-kosaki syndrome										
Author (references)	Takenouchi et al. (2015)	Takenouchi et al. (2016)	Motokawa et al. (2018)	Ishikawa et al. (2021)	Uehara et al. (2019)	Martinelli et al. (2018)	Bucciol et al. (2020)	Santoro et al. (2021)	Current report	Total
Age at diagnosis	18 years	22 years	12 years	15 years	4 years	15 years	25 years	7 years	9 months	
Pregnancy	normal	normal	Fetal hydrops	normal	normal	normal	normal	Abortion threats at first trimester	Premature uterine contractions	3 (33%)
			Pleural effusion							

Feeding disorders										
Feeding intolerance	no	yes, intestinal lymphangiectasia	no	no	no	no	yes	yes, poor sucking and swallowing	yes, poor sucking reflex, oral feeding disability	3 (33%)
Failure to thrive	yes	yes	yes	yes	no	no	yes	yes	yes	7 (78%)

Infections										
Respiratory	no	yes	yes	no	no	yes	yes, bronchiectasis, fatal pneumonia	no	Congenital pneumonia	5 (56%)
Gastrointestinal	no	no	no	no	no	no	no	no	Anterior gastric wall and intraperitoneal connective tissue	1 (11%)
Genitourinary	no	no	no	no	no	no	no	yes, Hydronephrosis	no	1 (11%)

Congenital anomalies										
Abnormal development of the internal organs	Persistent ductus arteriosus; Inguinal hernia	Retinal dysplasia with congenital detachment	no	no	no	Cardiac anomaly (unspecified)	no	Persistent ductus arteriosus; Ventricular septal defect	Atrial septal defect; Duplex pelvicalceal system of the right kidney; Small penis, scrotal hypoplasia; cryptorchidism	5 (56%)
Microcephaly	yes	yes	no	no	no data	no	yes	yes	no	4 (44%)
Short stature	yes	yes	no	yes	no data	yes	yes	yes	yes	7 (78%)
Camptodactyly	yes	yes	yes	yes	no	yes	yes	yes	yes	8 (89%)
Syndactyly	no	no	no	no	no	no	no	Cutaneous syndactyly of 2–3 toes bilaterally	Cutaneous syndactyly of 2–4 toes of the left and 2–3 toes of the right foot	2 (22%)

Facial features										
Arched eyebrows	yes	yes	no data	no	no data	yes	no	yes	yes	5 (56%)
Ptosis	yes	yes	yes	no	no data	no	no	no	yes	4 (44%)
Eversion of the lower eyelid	yes	yes	no data	no	no data	yes	yes	yes	no	5 (56%)
Synophrys	yes	yes	yes	yes	no data	no	no	yes	yes	6 (67%)
Exotropia	yes	yes	yes	no	no data	no	yes	yes	no	5 (56%)
Midface hypoplasia	yes	yes	yes	yes	no data	yes	no data	no	yes	6 (67%)
Short philtrum	yes	yes	no	yes	no data	smooth	no data	yes	no	4 (44%)
Thin upper lip	yes	yes	yes	no	no data	yes	no data	yes	yes	6 (67%)
Malocclusion	yes	yes	no data	no	no data	yes	yes	yes	no	5 (56%)

Psychomotor disability										
Speech	simple words	no	simple words	no data	no data	no data	no data	no data	Absent (not yet)	2 (22%)
Sensorineural hearing loss	yes	yes	yes	yes	yes	yes	yes	yes	yes	9 (100%)
Walking independency	4 years	3 years	3 years	no data	no data	no data	no data	no data	Absent (not yet)	3 (33%)
Neurological symptoms	tremor; ataxic gait	Nystagmus, agitation, sleep impairment	no	no	no	Seizures	no	no	Poor sucking reflex; axial hypotonia, peripheral spasticity	4 (44%)

MR imaging of the CNS										
Cerebellar atrophy	yes	no	no	no	yes	yes	yes, Dandy-Walker variant	no	Hypoplastic cerebellar vermis	5 (56%)
Corpus callosum hypoplasia	no	no	yes	no	no	no	no	no	no	1 (11%)
Ventriculomegaly	yes	yes	yes	no	yes	yes	yes	yes	yes	8 (89%)

Laboratory findings										
Macrothrombocytopenia	yes	yes	yes	yes	yes	yes	yes	intermittent	yes	9 (100%)
Antibody deficiency	no data	no data	yes	yes	no data	yes	yes	no data	yes	5 (56%)

The ever-increasing use of the next-generation sequencing (NGS) approach enabling uncovering molecular genetic diagnosis underpinning the clinical symptomatology has led to better recognition of variable *CDC42* gene alterations and the spectrum of clinical immunophenotypes. A variable degree of immunodeficiency has been primarily linked to the variants affecting the switch II domain in the *CDC42* gene (Takenouchi et al., 2015; Takenouchi et al., 2016; Martinelli et al., 2018; Motokawa et al., 2018; Uehara et al., 2019; Bucciol et al., 2020; Asiri et al., 2021; Ishikawa et al., 2021; Kashani et al., 2021; Santoro et al., 2021). In the reported patients, the antibody immune response ranged from normal referenced serum immunoglobulin levels to panhypogammaglobulinemia affecting the production of all immunoglobulin isotypes with low specific antibody response to diphtheria, tetanus, and mumps vaccine antigens. Antibody deficiency is a consequence of disrupted Cdc42 activity affecting actin cytoskeleton mobility, cellular trafficking, regulation of transcription, and proliferation which occur in multiple cell lineages in *CDC42* mutants, including the B and T cell compartments. Furthermore, in the actin cytoskeleton remodeling and cell polarity regulation, the Cdc42 GTPase activating interaction is required with the GTPase-binding domain of the Cdc42 effector, the Wiskott-Aldrich protein (WASP) that is essential for B and T cell differentiation (Tangye et al., 2019). The presently described patient has normal for age IgG, and IgM, decreased IgA levels, normal distribution of IgG subclasses, low antigen-specific anti-tetanus and anti-diphtheria antibodies, accompanied by lymphopenia and aberrant B and T lymph cell, primarily T helper cell development that might be underpinning the proneness to infections and immune dysregulation. Referring to the clinical reports, in 2022, disorders of the actin cytoskeleton affecting immunity have been included by the International Union of Immunological Societies Expert Committee in the updated classification of human inborn errors of immunity (Tangye et al., 2022).

In this report, we add feeding disorders as well as gastric and abdominal walls infection to the clinical symptomatology of the p.Tyr64Cys variant-related syndrome. Noteworthy, the aberrant immune response in our patient may be an important factor contributing to the chronic inflammation and malfunctioning of the gastrointestinal tract leading to a vicious circle of immunodeficiency with immune dysregulation and malnutrition with poor weight gaining, which, in turn, may exacerbate dysfunction of the immune system. This hypothesis may be supported by the master regulatory role of Cdc42 GTPase in maintaining the integration and immune homeostasis of the intestinal mucosa, and as a key player in the actin cytoskeleton dynamics, it controls mechanisms of inflammation, autoimmunity, and cancerogenesis. Intestinal cell proliferation, epithelial integrity, and cellular turnover as well as enterocyte extrusion and shedding are tightly controlled processes regulated by Cdc42-dependent actin cytoskeletal contractility and reorganization (Pradham et al., 2021; Ngo et al., 2022).

## The patient’s perspective

The patient’s perspective is largely dependent on the detection of the causative variant and establishing a definitive genetic diagnosis which primarily means an explanation for the multifaceted disease. Both for the family and leading physicians, it paves the way for future diagnostic and therapeutic interventions, shedding light on the expanded phenotype with immunodeficiency and gastrointestinal disorders. Prophylaxis against infections with azithromycin is legitimate as the first-line protective measure against respiratory tract infections. Administration of inactive vaccines needs to be recommended as an attempt to trigger the mounting of the immune memory yet the antigen-specific response in the patient may not be adequate (Szczawińska-Popłonyk et al., 2015). Regular monitoring is required by a multidisciplinary team including specialists in neurology, hematology, otorhinolaryngology, gastroenterology, cardiology, and nephrology, under the pediatric immunologist’s supervision. Further clinical observation is needed as other phenotypic features may appear and novel treatment modalities may be proposed according to the course of the disease. It is worth noting that the Rho GTPase Cdc42 lies downstream of the master regulator Ras, crucial for cell malignant transformation. The crosstalk between the Cdc42 molecule and the network of effectors in the immune system is increasingly recognized, and new therapeutic targets based on new regulator partners are expected, and their translation into the treatment strategies for cancer creating a future therapeutic perspective (El Masri and Delon, 2021).

Importantly, the everyday struggle with the child’s intellectual and motor disabilities, failure to thrive and feeding disorders, recurrent infections, increased risk of tumorigenesis, and frequent medical consultations is a disease burden for the patient’s relatives. To cope with the multisystemic syndrome of TKS, medical and social support is needed for the family to enhance undertaking positive health-promoting stimulating activities. Therefore, the *de novo* nature of the pathogenic *CDC42* variant has an important informative role for the family.

The ever-increasing progress in molecular genetic studies contributes to better definition and delineation of the Takenouchi-Kosaki syndrome. It is a multifaceted disease with complex phenotypic features and a broad dysmorphology, neurological, hematological, hormonal, respiratory as well as immunological symptomatology. In this report, we add gastrointestinal and feeding disorders to the spectrum of syndromic manifestations. Uncovering the causative pathogenic variant in the *CDC42* gene is of utmost importance for understanding the complex mutual genetic and phenotypic relationships and disease pathophysiology as well as for the informativeness of the patient’s family and the leading physician, contributing to diagnostic and therapeutic management and anticipating the prognosis.

## Data Availability

The datasets for this article are not publicly available due to concerns regarding participant/patient anonymity. Requests to access the datasets should be directed to the corresponding author.
